# Atypical Clinical Presentation of Hypothermia Induced by Intrathecal Morphine in a Patient Who Underwent Total Knee Arthroplasty Under Spinal Anesthesia: A Case Report

**DOI:** 10.7759/cureus.74615

**Published:** 2024-11-27

**Authors:** Denis Correa, Jeff Correa, Yamini Subramani, Yifan Zhang, Rowaida Hussein, Mahesh Nagappa

**Affiliations:** 1 Department of Anesthesia and Perioperative Medicine, Edmundston Regional Hospital, Edmundston, CAN; 2 Department of Anesthesiology and Perioperative Medicine, St. George's University School of Medicine, University Centre Grenada, West Indies, GRD; 3 Department of Anesthesia and Perioperative Medicine, London Health Sciences Centre, and St. Joseph Health Care, Lawson Health Research Institute, Schulich School of Medicine and Dentistry, Western University, London, CAN

**Keywords:** altered thermoregulation, hypothermia, intrathecal morphine, naloxone, spinal anesthesia

## Abstract

This case report describes a 62-year-old male who experienced paradoxical hypothermia following an elective total knee replacement (TKR) performed under spinal anesthesia with intrathecal morphine administration. The patient presented with significant hypothermia, defined as a body temperature below 35°C, approximately 45 minutes following the administration of anesthesia. The condition demonstrated resistance to conventional rewarming techniques. This condition required symptomatic treatment, leading to the administration of naloxone. After receiving two doses of naloxone, the patient’s temperature stabilized, and they experienced a reduction in diaphoresis, improved alertness, and less nausea. Given the scarce documentation on non-obstetric paradoxical hypothermic reactions to intrathecal morphine, this case highlights the critical need for careful temperature monitoring when administering spinal anesthesia with intrathecal opioids. Additionally, it highlights the unusual symptoms of hypothermia that can hinder diagnosis and treatment, stressing the need for increased awareness among anesthesiologists to achieve the best possible patient outcomes.

## Introduction

We present a case of early-onset paradoxical signs and symptoms associated with perioperative hypothermia (temperature below 35ºC) observed in the operating room after elective total knee arthroplasty (TKA) performed under spinal anesthesia with morphine.

Intrathecal morphine utilized for post-operative pain management offers optimal and sustained analgesia and has been in practice since 1980 [1]. The benefits of intrathecal morphine encompass its opioid-sparing effects during the postoperative period, its targeted action within the central nervous system, and the prolonged duration of action facilitated by its interaction with opioid receptors in the spinal cord [1]. In addition to the commonly observed side effects, including pruritus, nausea, vomiting, and delayed respiratory depression, spinal anesthesia with morphine is associated with a mild reduction in body temperature, not falling below 35.8ºC. The observed decrease in temperature, which averages 0.76ºC, can be attributed to the sympatholytic effect of local anesthetics. Additionally, there is a further decline of 0.35ºC resulting from the administration of morphine. Consequently, this results in an overall temperature reduction of 1.1ºC, which is attributable to vasodilation and the attenuation of the shivering reflex. Typically, this reduction in temperature holds minimal clinical significance and is addressed through rewarming measures [2].

Over the past three decades, a limited number of cases have been documented, predominantly within obstetric populations related to cesarean sections [3-8]. Only three non-obstetric cases have been reported: one concerning orthopedic surgery, another involving a skin graft, and the final case on the resection of a pelvic tumor in a teenage boy [2,9,10]. In all instances, the manifestation of atypical symptoms associated with hypothermia was observed in the recovery room approximately two to three hours following the administration of spinal anesthesia.

The initial instance of severe hypothermia (less than 34ºC) linked to intrathecal morphine was documented in 1992 by Kosai et al. [9]. This report encompasses nine case reports (involving 10 patients) available in English and foreign language literature. Numerous case reports illustrate the occurrence of persistent and profound hypothermia following spinal anesthesia administered with intrathecal morphine within the obstetric population [11]. This condition is resistant to rewarming efforts, typically manifests two to three hours post spinal anesthesia, and tends to resolve within six hours.

Among the most intriguing phenomena are the paradoxical and atypical signs and symptoms associated with subjective warmth, such as feeling excessively hot, profuse sweating and restlessness occurring in hypothermic conditions [4]. These manifestations may be linked to several life-threatening conditions and can serve as distracting symptoms during the diagnosis of hypothermia [10]. The patient’s safety and optimal clinical outcomes must consider the early warning signs of hypothermia induced by spinal morphine and underscore the significance of body temperature monitoring during regional anesthesia. This practice is frequently overlooked due to accessibility issues. Due to the association of hypothermia with unfavorable clinical outcomes as well as psychological disturbances in patients, it is imperative to promptly recognize the atypical or paradoxical warning signs and symptoms of hypothermia induced by intrathecal morphine. This case report delineates the occurrence of persistent hypothermia after spinal anesthesia, utilizing intrathecal morphine for TKA, which resolved following the administration of naloxone.

## Case presentation

Written consent from the patient was obtained before the publication of the case report. This case report followed the CAse REport (CARE) guidelines [12]. A 62-year-old male patient, weighing 119.7 kg and measuring 186 cm, with a body mass index (BMI) of 34.6 kg/m^2^, presents a physical status classified as American Society of Anesthesiologists (ASA) 2. The patient was a former smoker and utilized opioids (morphine sulfate immediate release 5 mg) as needed, in addition to Celebrex for pain management. He underwent an elective TKA procedure at the Edmundston Regional Hospital, New Brunswick.

The patient’s medical history indicated an allergic reaction to penicillin and a background of opioid use on an as-needed basis, in addition to a history of smoking; however, no other significant medical issues were documented. The routine blood work results were reported as normal. The patient's surgical history was deemed inconsequential, except for the knee arthroscopy performed under general anesthesia, which was conducted without any complications. Furthermore, the patient's medical records had no documented spinal anesthesia history.

On the morning of the surgical procedure, at 06:30, the patient’s vital signs were documented and found to be within normal ranges. The heart rate was measured at 81 beats per minute, and the blood pressure was recorded at 144/66 mmHg. The oxygen saturation level was 98%. The respiratory rate was 16 breaths per minute, and the body temperature was 36.6°C.

After intravenous access was established in the holding area, the patient was transferred to the operating room at 09:40. Upon arrival, standard monitoring protocols were implemented, and the patient was positioned in a sitting posture for spinal anesthesia. The patient received mild sedation with 2 mg of midazolam and 50 µg of fentanyl.

At 10:07, spinal anesthesia was administered utilizing a 25G Whitacre needle, employing a midline approach at the L3-L4 interspace. A total of 2 ml of bupivacaine at a concentration of 0.75% (15 mg), 300 µg of morphine, and 20 µg of fentanyl were utilized during the procedure. The patient was positioned in the supine orientation, and the extent of sensory blockade at the T6 level was verified. Routine monitoring of vital signs was conducted, except for body temperature. A forced warming blanket and a warm intravenous ringer's lactate were utilized, and oxygen was administered at a flow rate of 4 liters per minute via a mask, accompanied by end-tidal carbon dioxide monitoring. Propofol sedation was initiated at a rate of 150 µg/kg/min. Additionally, 2 grams of cefazolin were administered intravenously before the tourniquet inflation.

The surgical procedure commenced at 10:25. By 10:50, approximately 25 minutes later and 43 minutes after the administration of a subarachnoid block, the patient began to articulate feelings of heat, sweat, nausea, and drowsiness and was subsequently observed to be restless. Vital signs were recorded, indicating a heart rate fluctuating between 56 and 92 beats per minute, stable blood pressure averaging 126/50 to 138/64 mmHg, hemoglobin oxygen saturation levels ranging from 96% to 100%, and a respiratory rate of 14 to 18 beats per minute (bpm), accompanied by a normal electrocardiogram assessment.

As an initial measure, we endeavored to ascertain the underlying causes and managed them symptomatically by ceasing the forced warming and infusion of propofol. Ondansetron 4 mg and metoclopramide 10 mg were administered, partially alleviating nausea; however, other symptoms remained unaltered. The procedure proceeded with reassurances and intermittent propofol administration. The surgical procedure was concluded at 12:15, and the patient was subsequently transferred to the post-anesthesia care unit (PACU) at 12:25.

The patient was observed to be drowsy yet easily arousable in the PACU. The vital signs recorded included a heart rate ranging from 50 to 60 bpm, blood pressure measurements between 120/60 and 140/75 mmHg, and an oxygen saturation level (SpO_2_) of 95% while receiving 4 liters of supplemental oxygen. The respiratory rate was noted to be between 14 and 16 breaths per minute, and the rectal temperature was measured at 34.7ºC at 12:35. 

The patient persistently exhibited symptoms of hyperthermia, including diaphoresis and nausea, and remained hypothermic despite the application of heated blankets. Vital signs and electrocardiogram results displayed no abnormalities. All postoperative investigative reports, including troponin levels and blood gas analysis, returned normal values.

The patient received treatment in the PACU for two hours and 20 minutes, specifically from 12:25 to 14:45. During this period, forced warming blankets, intravenous (IV) fluids, and supplemental oxygen were administered; however, no noticeable improvement was observed, and the patient's temperature remained at 34.7ºC.

The underlying cause of the nonresponsive hypothermia, nausea, sweating, and drowsiness remains unidentified, except for intrathecal morphine administration. At 14:55 hours, 200 micrograms of naloxone were administered. The patient demonstrated notable signs of improvement, displaying increased alertness, a reduction in nausea, and an elevation in temperature to 35.1ºC; however, perspiration persisted. After a 15-minute interval, an additional 200 micrograms of naloxone were administered. Subsequently, the temperature rose significantly to 35.4ºC, coinciding with the cessation of perspiration and the absence of nausea. 

In the PACU, a literature search identified a series of case reports detailing instances of hypothermia in parturients following cesarean sections performed under spinal anesthesia in conjunction with the administration of lorazepam and midazolam [3,13]. Consequently, a sublingual dose of 1 mg of lorazepam was administered to the patient. The patient was subsequently transferred to the ward after administering a femoral nerve block utilizing 20 ml of 0.25% ropivacaine at 15:55. The vital signs were within normal limits, and the cutaneous temperature measured 35.8ºC.

On the surgical floor at 16:07, the patient’s temperature was recorded at 36.1ºC, with a heart rate of 57 beats per minute, blood pressure measuring 148/57 mmHg, and oxygen saturation at 94%. Subsequently, at 17:00, the temperature increased slightly to 36.4ºC, while all other vital signs remained stable and within normal limits. Other than feelings of anxiety and restlessness on postoperative day one, the patient recovered uneventfully.

## Discussion

While there exist only three non-obstetric case reports (TKA, skin grafting, and pelvic mass resection) involving male patients, intrathecal morphine has been extensively utilized over the past three decades for various surgical procedures across both genders. Therefore, it would be reasonable to anticipate the occurrence of severe hypothermia and associated atypical symptoms in male patients, apart from those undergoing cesarean sections [2,9,10].

The precise mechanism through which intrathecal morphine induces this effect remains incompletely understood. Several authors have demonstrated that spinal morphine has exacerbated the hypothermic response induced by spinal local anesthetics in pregnant women following cesarean sections, as well as in three additional non-obstetric patients [14]. All patients reported experiencing nausea and a sensation of warmth while simultaneously exhibiting diaphoresis and hypothermia [15]. Body temperature regulation constitutes a complex and sophisticated process characterized by precise control within mere tenths of a degree. The spinal cord, brain, and hypothalamus collaboratively integrate signals from peripheral temperature receptors. This leads to autonomic responses that induce capillary vasodilation, promote sweating in elevated temperatures, and stimulate vasoconstriction and shivering in lower temperatures. 

The primary initial contribution to heat loss arises from the redistribution of heat from the core to the periphery, resulting from vasodilation associated with sympathectomy induced by the spinal block and propofol sedation. This phenomenon is most pronounced during the initial 30 to 60 minutes and is contingent upon the degree of the block. The second mechanism pertains to the loss of thermoregulatory vasoconstriction and shivering below the level of the block. Finally, the absence of tonic cold signals from the legs, resulting from spinal blockade, is interpreted by the central controller, namely the hypothalamus, as selective warmth [16].

The manifestation of paradoxical symptoms in our patient is likely attributable to the cephaloid dissemination of intrathecal morphine, which is hydrophilic. Alongside the usual hypothermic effects of spinal local anesthetics and intrathecal morphine, the hypothalamus's involvement from morphine's rostral spread, sweating's evaporation cooling, and the application of cooling techniques (such as cold compresses and fans) likely contributed to the overall drop in temperature, compounded by misleading paradoxical hypothermic symptoms [7].

The ramifications of perioperative hypothermia encompass coagulopathy, augmented blood loss, an elevated rate of surgical site infections, disturbances in cardiac rhythm, myocardial ischemia, an extended duration in the PACU and hospital, patient discomfort, and escalating costs [17]. The ongoing monitoring of core temperature, specifically nasopharyngeal and esophageal temperatures, during spinal anesthesia presents significant challenges due to the patient's conscious state. In this context, the most appropriate estimation of core body temperature is achieved through sublingual or aural canal measurement; however, there remains debate concerning the precision of these methods.

A recent survey shows body temperature monitoring during regional anesthesia is infrequently practiced. This is attributed to the absence of malignant hyperthermia and the lack of a convenient and reliable site for inserting a temperature probe. A recent survey concluded that only 33% of practicing anesthesiologists regularly monitor the temperature of patients receiving regional anesthesia, thereby placing them at considerable risk for hypothermia, which necessitates vigilant monitoring [18]. Hypothermia is frequently unrecognized and untreated in patients undergoing regional anesthesia, even in cases where the patients remain conscious. These patients are typically reluctant to report cold sensations due to the “apparent” warming input originating from the body regions below the level of neural blockade. Frequently, the initial indication of hypothermia presents postoperatively as the spinal blockade begins to dissipate [19].

Body temperature monitoring is not routinely implemented during regional anesthesia, resulting in the unrecognized occurrence of perioperative hypothermia among a considerable number of patients [20]. Given that hypothermia is linked to adverse clinical outcomes, including infection, bleeding, cardiac injury, discomfort, and shivering, it is imperative to identify the clinical warning signs and symptoms of hypothermia. Finally, meticulous body temperature monitoring and regulation in at-risk patients are essential.

## Conclusions

This case report underscores the early manifestation of warning paradoxical signs of hypothermia resulting from the administration of intrathecal morphine during TKA. The simultaneous administration of intrathecal morphine significantly modifies the standard thermoregulatory response. Paradoxically, this may manifest as sweat and hyperthermia and be accompanied by low temperature, potentially resulting in erroneous and misleading diagnoses and management measures that could lead to adverse outcomes.

Although continuous monitoring of core temperature (nasopharyngeal and esophageal) is not routinely practiced in regional anesthesia due to the patient's conscious state, it is prudent to implement it. This approach allows for the early recognition and comprehension of the clinical response and presentation, facilitating the initiation of appropriate patient care.
